# (Methyldiphenyl­phospho­ranylidene)­ammonium chloride

**DOI:** 10.1107/S1600536809020698

**Published:** 2009-06-06

**Authors:** Cintya Valerio-Cárdenas, Luis Ortiz-Frade, Jean-Michel Grévy M.

**Affiliations:** a Centro de Investigaciones Químicas, Universidad Autónoma del Estado de Morelos, Av. Universidad 1001, Col. Chamilpa Cuernavaca, Morelos 62210, Mexico; b Electrochemistry Department, Centro de Investigación y Desarrollo Tecnológico en Electroquímica SC, Parque Tecnológico Querétaro, Sanfandila, Pedro de Escobedo, CP 76703, Querétaro, Mexico

## Abstract

The title compound, C_13_H_15_NP^+^·Cl^−^, was obtained by hydrolysis of the *N*-trimethysilyl derivative of methydiphenyl­imino­phosphine. The dihedral angle between the phenyl rings in the cation is 61.5 (3)°. In the crystal structure, inter­molecular N—H⋯Cl hydrogen bonds links the two components, forming a centrosymmetric 2 + 2 aggregate.

## Related literature

For imino­phosphines, see: Appel & Hauss (1960 ▶); Hitchcock *et al.* (1999 ▶). For a related structure, see: Clegg & Bleasdale (1994 ▶).
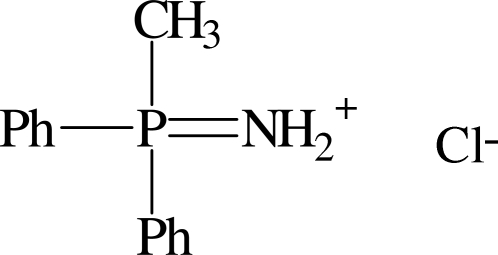


## Experimental

### 

#### Crystal data

C_13_H_15_NP^+^·Cl^−^
*M* *_r_* = 251.68
Monoclinic, 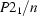

*a* = 9.4760 (14) Å
*b* = 11.8411 (18) Å
*c* = 11.8382 (18) Å
β = 107.773 (2)°
*V* = 1264.9 (3) Å^3^
*Z* = 4
Mo *K*α radiation
μ = 0.4 mm^−1^
*T* = 298 K
0.54 × 0.37 × 0.28 mm

#### Data collection

Brucker SMART 6000 CCD area-detector diffractometer
Absorption correction: none
7223 measured reflections
2226 independent reflections
2089 reflections with *I* > 2σ(*I*)
*R*_int_ = 0.026

#### Refinement

*R*[*F* ^2^ > 2σ(*F* ^2^)] = 0.036
*wR*(*F* ^2^) = 0.089
*S* = 1.14
2226 reflections
152 parameters
H atoms treated by a mixture of independent and constrained refinement
Δρ_max_ = 0.48 e Å^−3^
Δρ_min_ = −0.25 e Å^−3^

### 

Data collection: *SMART* (Bruker, 2001 ▶); cell refinement: *SAINT* (Bruker, 2001 ▶); data reduction: *SAINT*; program(s) used to solve structure: *SHELXS86* (Sheldrick, 2008 ▶); program(s) used to refine structure: *SHELXL97* (Sheldrick, 2008 ▶); molecular graphics: *ORTEP-3* (Farrugia, 1997 ▶); software used to prepare material for publication: *WinGX* (Farrugia, 1999 ▶).

## Supplementary Material

Crystal structure: contains datablocks I, global. DOI: 10.1107/S1600536809020698/is2426sup1.cif

Structure factors: contains datablocks I. DOI: 10.1107/S1600536809020698/is2426Isup2.hkl

Additional supplementary materials:  crystallographic information; 3D view; checkCIF report

## Figures and Tables

**Table 1:** Hydrogen-bond geometry (Å, °)

*D*—H⋯*A*	*D*—H	H⋯*A*	*D*⋯*A*	*D*—H⋯*A*
N1—H100⋯Cl1^i^	0.81 (3)	2.37 (3)	3.181 (2)	176 (2)
N1—H101⋯Cl1^ii^	0.84 (3)	2.35 (3)	3.173 (2)	167 (2)

Symmetry codes: (i) 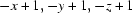; (ii) 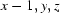.

**Fig. 1.**
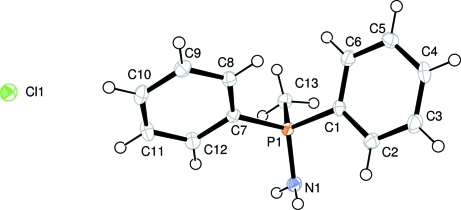
The molecular structure of the title compound, with atom labels and 50% probability displacement ellipsoids for non-H atoms.

**Fig. 2.**
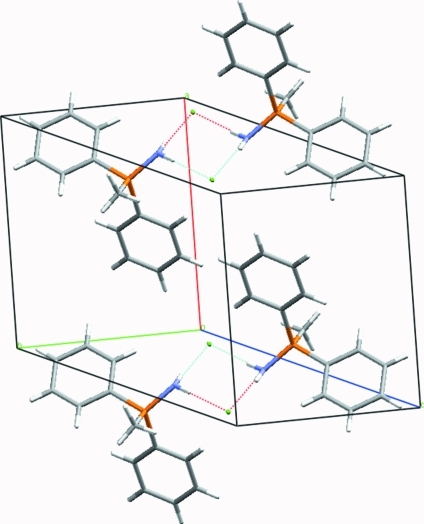
A packing diagram of the title compound, showing molecules connected by N—H···Cl hydrogen bonds (dashed lines).

## supplementary crystallographic information

### Comment

The synthesis and coordination chemistry of iminophosphine ligands have attracted much interest during the past two decades owing to the potential applications of their complexes in catalysis (Hitchcock *et al.*, 1999). The procedures previously reported for the synthesis of [*R*_3_PNH_2_]^+^ cations involve the reaction of trialkylphosphines with hazardous chemicals such as chloramine (Appel & Hauss, 1960) or hydrogen azide (Clegg & Bleasdale, 1994). Here we report the structure of aminophosphonium salt, (I), obtained by reaction of the *N*-trimethylsylil derivative of methyldiphenyliminophosphine with a mixture of MgCl_2 _and pyridine of technical grade.

The title compound, C_13_H_15_NPCl, is formed by cations [(C_6_H_5_)_2_CH_3_P=NH_2_]^+^ and anions Cl^-^. In this cation (Fig. 1) a pseudo-tetrahedral and a trigonal planar geometries are observed around the P atom and the N atom, respectively. In the crystal structure, intermolecular N—H···Cl hydrogen bonds (Fig. 2) link two cations [(C_6_H_5_)_2_CH_3_P=NH_2_]^+^ through two anions Cl^- ^, generating a distorted square arrangement along the *a* axis.

### Experimental

The title compound was isolated from the reaction mixture of CH_3_(Ph_2_)P=N(SiMe_3_) and MgCl_2_ in a 1:1 molar ratio in pyridine, and was crystallized from pyridine.

### Refinement

H atoms bound to N1 were located in a difference Fourier map and refined freely. Other atoms were positioned geometrically and refined as riding model, with C—H = 0.93 or 0.96 Å, and with *U*_iso_(H) = 1.2*U*_eq_(C, phenyl) or 1.5*U*_eq_(C, methyl).

### Crystal data

**Table:**

C_13_H_15_NP^+^·Cl^−^	*F*(000) = 528
*M**_r_* = 251.68	*D*_x_ = 1.322 Mg m^−^^3^
Monoclinic, *P*2_1_/*n*	Mo *K*α radiation, λ = 0.71073 Å
Hall symbol: -P 2yn	Cell parameters from 7223 reflections
*a* = 9.4760 (14) Å	θ = 2.4–25°
*b* = 11.8411 (18) Å	µ = 0.4 mm^−^^1^
*c* = 11.8382 (18) Å	*T* = 298 K
β = 107.773 (2)°	Prism, colorless
*V* = 1264.9 (3) Å^3^	0.54 × 0.37 × 0.28 mm
*Z* = 4	

### Data collection

**Table:**

Brucker 6000 CCD area-detector diffractometer	2089 reflections with *I* > 2σ(*I*)
Radiation source: sealed tube	*R*_int_ = 0.026
graphite	θ_max_ = 25°, θ_min_ = 2.4°
φ and ω scans	*h* = −11→7
7223 measured reflections	*k* = −14→14
2226 independent reflections	*l* = −14→13

### Refinement

**Table:**

Refinement on *F*^2^	Primary atom site location: structure-invariant direct methods
Least-squares matrix: full	Secondary atom site location: difference Fourier map
*R*[*F*^2^ > 2σ(*F*^2^)] = 0.036	Hydrogen site location: inferred from neighbouring sites
*wR*(*F*^2^) = 0.089	H atoms treated by a mixture of independent and constrained refinement
*S* = 1.14	*w* = 1/[σ^2^(*F*_o_^2^) + (0.0386*P*)^2^ + 0.8702*P*] where *P* = (*F*_o_^2^ + 2*F*_c_^2^)/3
2226 reflections	(Δ/σ)_max_ < 0.001
152 parameters	Δρ_max_ = 0.48 e Å^−^^3^
0 restraints	Δρ_min_ = −0.25 e Å^−^^3^

### Special details

**Table:**

Geometry. All e.s.d.'s (except the e.s.d. in the dihedral angle between two l.s. planes) are estimated using the full covariance matrix. The cell e.s.d.'s are taken into account individually in the estimation of e.s.d.'s in distances, angles and torsion angles; correlations between e.s.d.'s in cell parameters are only used when they are defined by crystal symmetry. An approximate (isotropic) treatment of cell e.s.d.'s is used for estimating e.s.d.'s involving l.s. planes.
Refinement. Refinement of *F*^2^ against ALL reflections. The weighted *R*-factor *wR* and goodness of fit *S* are based on *F*^2^, conventional *R*-factors *R* are based on *F*, with *F* set to zero for negative *F*^2^. The threshold expression of *F*^2^ > σ(*F*^2^) is used only for calculating *R*-factors(gt) *etc*. and is not relevant to the choice of reflections for refinement. *R*-factors based on *F*^2^ are statistically about twice as large as those based on *F*, and *R*- factors based on ALL data will be even larger.

### Fractional atomic coordinates and isotropic or equivalent isotropic displacement parameters (Å^2^)

**Table:**

	*x*	*y*	*z*	*U*_iso_*/*U*_eq_	
P1	0.20314 (5)	0.33420 (4)	0.71372 (4)	0.01414 (15)	
N1	0.06353 (19)	0.39343 (16)	0.61815 (16)	0.0187 (4)	
Cl1	0.91197 (5)	0.62714 (4)	0.64308 (4)	0.02031 (15)	
C1	0.1596 (2)	0.31208 (16)	0.84887 (17)	0.0162 (4)	
C2	0.0407 (2)	0.36780 (17)	0.86978 (19)	0.0212 (5)	
H2	−0.0166	0.4178	0.8138	0.025*	
C3	0.0086 (3)	0.34818 (18)	0.97476 (19)	0.0249 (5)	
H3	−0.0703	0.3858	0.9893	0.03*	
C4	0.0922 (2)	0.27361 (18)	1.05784 (18)	0.0224 (5)	
H4	0.0693	0.2606	1.1277	0.027*	
C5	0.2106 (2)	0.21799 (18)	1.03713 (18)	0.0232 (5)	
H5	0.2672	0.1678	1.0932	0.028*	
C6	0.2447 (2)	0.23704 (17)	0.93316 (18)	0.0205 (4)	
H6	0.3244	0.1998	0.9195	0.025*	
C7	0.3717 (2)	0.41427 (15)	0.74335 (17)	0.0153 (4)	
C8	0.4749 (2)	0.42223 (17)	0.85534 (17)	0.0184 (4)	
H8	0.4571	0.387	0.9199	0.022*	
C9	0.6046 (2)	0.48279 (17)	0.87060 (18)	0.0213 (5)	
H9	0.6731	0.489	0.9457	0.026*	
C10	0.6325 (2)	0.53406 (17)	0.77462 (19)	0.0219 (5)	
H10	0.7204	0.5737	0.7851	0.026*	
C11	0.5301 (2)	0.52647 (18)	0.66312 (19)	0.0243 (5)	
H11	0.5491	0.5611	0.5988	0.029*	
C12	0.3994 (2)	0.46749 (18)	0.64691 (18)	0.0214 (5)	
H12	0.33	0.4633	0.572	0.026*	
C13	0.2304 (2)	0.20247 (16)	0.65085 (18)	0.0190 (4)	
H13A	0.3207	0.1686	0.6992	0.029*	
H13B	0.2365	0.2146	0.5723	0.029*	
H13C	0.1487	0.1532	0.6472	0.029*	
H100	0.068 (3)	0.391 (2)	0.550 (2)	0.029*	
H101	0.033 (3)	0.456 (2)	0.637 (2)	0.029*	

### Atomic displacement parameters (Å^2^)

**Table:**

	*U*^11^	*U*^22^	*U*^33^	*U*^12^	*U*^13^	*U*^23^
P1	0.0128 (3)	0.0156 (3)	0.0142 (3)	−0.00095 (19)	0.0046 (2)	−0.00070 (18)
N1	0.0175 (9)	0.0236 (9)	0.0157 (9)	0.0019 (7)	0.0059 (7)	0.0003 (7)
Cl1	0.0202 (3)	0.0240 (3)	0.0178 (3)	0.0003 (2)	0.0075 (2)	−0.00091 (19)
C1	0.0158 (10)	0.0173 (10)	0.0159 (10)	−0.0044 (8)	0.0053 (8)	−0.0025 (8)
C2	0.0194 (11)	0.0199 (10)	0.0253 (11)	0.0020 (8)	0.0082 (9)	0.0014 (8)
C3	0.0240 (12)	0.0271 (11)	0.0285 (12)	0.0021 (9)	0.0154 (10)	−0.0013 (9)
C4	0.0244 (12)	0.0281 (11)	0.0184 (10)	−0.0092 (9)	0.0123 (9)	−0.0037 (8)
C5	0.0217 (11)	0.0292 (11)	0.0174 (10)	−0.0005 (9)	0.0039 (8)	0.0047 (9)
C6	0.0170 (11)	0.0247 (11)	0.0211 (10)	0.0004 (9)	0.0075 (8)	0.0003 (8)
C7	0.0141 (10)	0.0135 (9)	0.0186 (10)	0.0007 (8)	0.0056 (8)	−0.0021 (7)
C8	0.0176 (10)	0.0206 (10)	0.0177 (10)	−0.0002 (8)	0.0065 (8)	0.0012 (8)
C9	0.0173 (11)	0.0237 (11)	0.0204 (10)	−0.0021 (8)	0.0024 (8)	−0.0032 (8)
C10	0.0178 (11)	0.0211 (10)	0.0289 (11)	−0.0060 (8)	0.0101 (9)	−0.0050 (9)
C11	0.0286 (12)	0.0259 (11)	0.0218 (11)	−0.0067 (9)	0.0127 (9)	0.0011 (9)
C12	0.0212 (11)	0.0249 (11)	0.0171 (10)	−0.0045 (9)	0.0044 (8)	−0.0011 (8)
C13	0.0202 (11)	0.0180 (10)	0.0202 (10)	−0.0006 (8)	0.0083 (8)	−0.0034 (8)

### Geometric parameters (Å, °)

**Table:**

P1—N1	1.6150 (18)	C6—H6	0.93
P1—C13	1.781 (2)	C7—C8	1.390 (3)
P1—C1	1.789 (2)	C7—C12	1.397 (3)
P1—C7	1.798 (2)	C8—C9	1.386 (3)
N1—H100	0.81 (3)	C8—H8	0.93
N1—H101	0.84 (3)	C9—C10	1.383 (3)
C1—C2	1.391 (3)	C9—H9	0.93
C1—C6	1.395 (3)	C10—C11	1.382 (3)
C2—C3	1.387 (3)	C10—H10	0.93
C2—H2	0.93	C11—C12	1.384 (3)
C3—C4	1.378 (3)	C11—H11	0.93
C3—H3	0.93	C12—H12	0.93
C4—C5	1.386 (3)	C13—H13A	0.96
C4—H4	0.93	C13—H13B	0.96
C5—C6	1.383 (3)	C13—H13C	0.96
C5—H5	0.93		
			
N1—P1—C13	106.26 (10)	C1—C6—H6	120
N1—P1—C1	109.06 (10)	C8—C7—C12	119.72 (18)
C13—P1—C1	110.36 (9)	C8—C7—P1	123.15 (15)
N1—P1—C7	113.38 (9)	C12—C7—P1	117.10 (14)
C13—P1—C7	108.05 (9)	C9—C8—C7	119.82 (19)
C1—P1—C7	109.67 (9)	C9—C8—H8	120.1
P1—N1—H100	113.4 (18)	C7—C8—H8	120.1
P1—N1—H101	117.9 (17)	C10—C9—C8	120.29 (19)
H100—N1—H101	114 (2)	C10—C9—H9	119.9
C2—C1—C6	119.87 (18)	C8—C9—H9	119.9
C2—C1—P1	120.66 (15)	C11—C10—C9	120.08 (19)
C6—C1—P1	119.46 (15)	C11—C10—H10	120
C3—C2—C1	119.44 (19)	C9—C10—H10	120
C3—C2—H2	120.3	C10—C11—C12	120.23 (19)
C1—C2—H2	120.3	C10—C11—H11	119.9
C4—C3—C2	120.7 (2)	C12—C11—H11	119.9
C4—C3—H3	119.6	C11—C12—C7	119.85 (18)
C2—C3—H3	119.6	C11—C12—H12	120.1
C3—C4—C5	119.91 (19)	C7—C12—H12	120.1
C3—C4—H4	120	P1—C13—H13A	109.5
C5—C4—H4	120	P1—C13—H13B	109.5
C6—C5—C4	120.11 (19)	H13A—C13—H13B	109.5
C6—C5—H5	119.9	P1—C13—H13C	109.5
C4—C5—H5	119.9	H13A—C13—H13C	109.5
C5—C6—C1	119.94 (19)	H13B—C13—H13C	109.5
C5—C6—H6	120		
			
N1—P1—C1—C2	−15.77 (19)	N1—P1—C7—C8	142.46 (17)
C13—P1—C1—C2	−132.14 (17)	C13—P1—C7—C8	−100.05 (18)
C7—P1—C1—C2	108.93 (17)	C1—P1—C7—C8	20.29 (19)
N1—P1—C1—C6	163.41 (16)	N1—P1—C7—C12	−39.42 (19)
C13—P1—C1—C6	47.04 (19)	C13—P1—C7—C12	78.07 (17)
C7—P1—C1—C6	−71.89 (18)	C1—P1—C7—C12	−161.59 (15)
C6—C1—C2—C3	0.2 (3)	C12—C7—C8—C9	0.0 (3)
P1—C1—C2—C3	179.35 (16)	P1—C7—C8—C9	178.04 (15)
C1—C2—C3—C4	−0.5 (3)	C7—C8—C9—C10	−0.9 (3)
C2—C3—C4—C5	0.5 (3)	C8—C9—C10—C11	0.9 (3)
C3—C4—C5—C6	−0.1 (3)	C9—C10—C11—C12	−0.1 (3)
C4—C5—C6—C1	−0.2 (3)	C10—C11—C12—C7	−0.8 (3)
C2—C1—C6—C5	0.2 (3)	C8—C7—C12—C11	0.9 (3)
P1—C1—C6—C5	−179.01 (16)	P1—C7—C12—C11	−177.32 (16)

### Hydrogen-bond geometry (Å, °)

**Table:**

*D*—H···*A*	*D*—H	H···*A*	*D*···*A*	*D*—H···*A*
N1—H100···Cl1^i^	0.81 (3)	2.37 (3)	3.181 (2)	176 (2)
N1—H101···Cl1^ii^	0.84 (3)	2.35 (3)	3.173 (2)	167 (2)

Symmetry codes: (i) −*x*+1, −*y*+1, −*z*+1; (ii) *x*−1, *y*, *z*.
